# Cryptococcal fungemia and *Mycobacterium haemophilum* cellulitis in a patient receiving ruxolitinib: a case report and literature review

**DOI:** 10.1186/s12879-020-05703-2

**Published:** 2021-01-07

**Authors:** Naruemit Sayabovorn, Piriyaporn Chongtrakool, Methee Chayakulkeeree

**Affiliations:** 1 Division of Infectious Diseases and Tropical Medicine, Department of Medicine, Faculty of Medicine Siriraj Hospital, Mahidol University, 2 Wanglang Road, Bangkoknoi, Bangkok, 10700 Thailand; 2 Department of Microbiology, Faculty of Medicine Siriraj Hospital, Mahidol University, Bangkok, Thailand

**Keywords:** Ruxolitinib, Myelofibrosis, Cryptococcosis, *Cryptococcus*, *Mycobacterium haemophilum*

## Abstract

**Background:**

Ruxolitinib is a novel oral Janus kinase inhibitor that is used for treatment of myeloproliferative diseases. It exhibits potent anti-inflammatory and immunosuppressive effects, and may increase the risk of opportunistic infections. Here, we report a rare case of *Cryptococcus neoformans* and *Mycobacterium haemophilum* coinfection in a myelofibrosis patient who was receiving ruxolitinib.

**Case presentation:**

A 70-year-old Thai man who was diagnosed with JAK2V617F-mutation-positive primary myelofibrosis had been treated with ruxolitinib for 4 years. He presented with cellulitis at his left leg for 1 week. Physical examination revealed fever, dyspnea, desaturation, and sign of inflammation on the left leg and ulcers on the right foot. Blood cultures showed positive for *C. neoformans*. He was prescribed intravenous amphotericin B deoxycholate with a subsequent switch to liposomal amphotericin B due to the development of acute kidney injury. He developed new onset of fever after 1 month of antifungal treatment, and the lesion on his left leg had worsened. Biopsy of that skin lesion was sent for mycobacterial culture, and the result showed *M. haemophilum*. He was treated with levofloxacin, ethambutol, and rifampicin; however, the patient eventually developed septic shock and expired.

**Conclusions:**

This is the first case of *C. neoformans* and *M. haemophilum* coinfection in a patient receiving ruxolitinib treatment. Although uncommon, clinicians should be aware of the potential for multiple opportunistic infections that may be caused by atypical pathogens in patients receiving ruxolitinib.

## Background

Ruxolitinib is a novel biologic agent used for the treatment of myeloproliferative diseases, that inhibits Janus kinase 1 (JAK1) and Janus kinase 2 (JAK2). Although it is effective and had a survival benefit in such patients, this immunosuppressive agent may increase the risk of opportunistic infections. However, reports related to its infectious complications is limited. Previous studies reported bacteria, *Mycobacterium tuberculosis*, *Cryptococcus neoformans*, *Pneumocystis jirovecii*, herpes simplex, and varicella zoster virus to be etiologic agents that were isolated from patients receiving ruxolitinib [1]. Furthermore, no previous case has been reported of *Mycobacterium haemophilum* infection or concurrent infection by two pathogens in a patient receiving ruxolitinib treatment. Here, we report a ruxolitinib-treated patient with rare infectious complication of *C. neoformans* fungemia who was co-infected with *M. haemophilum* cellulitis at his left leg.

## Case presentation

**Fig. 1:**
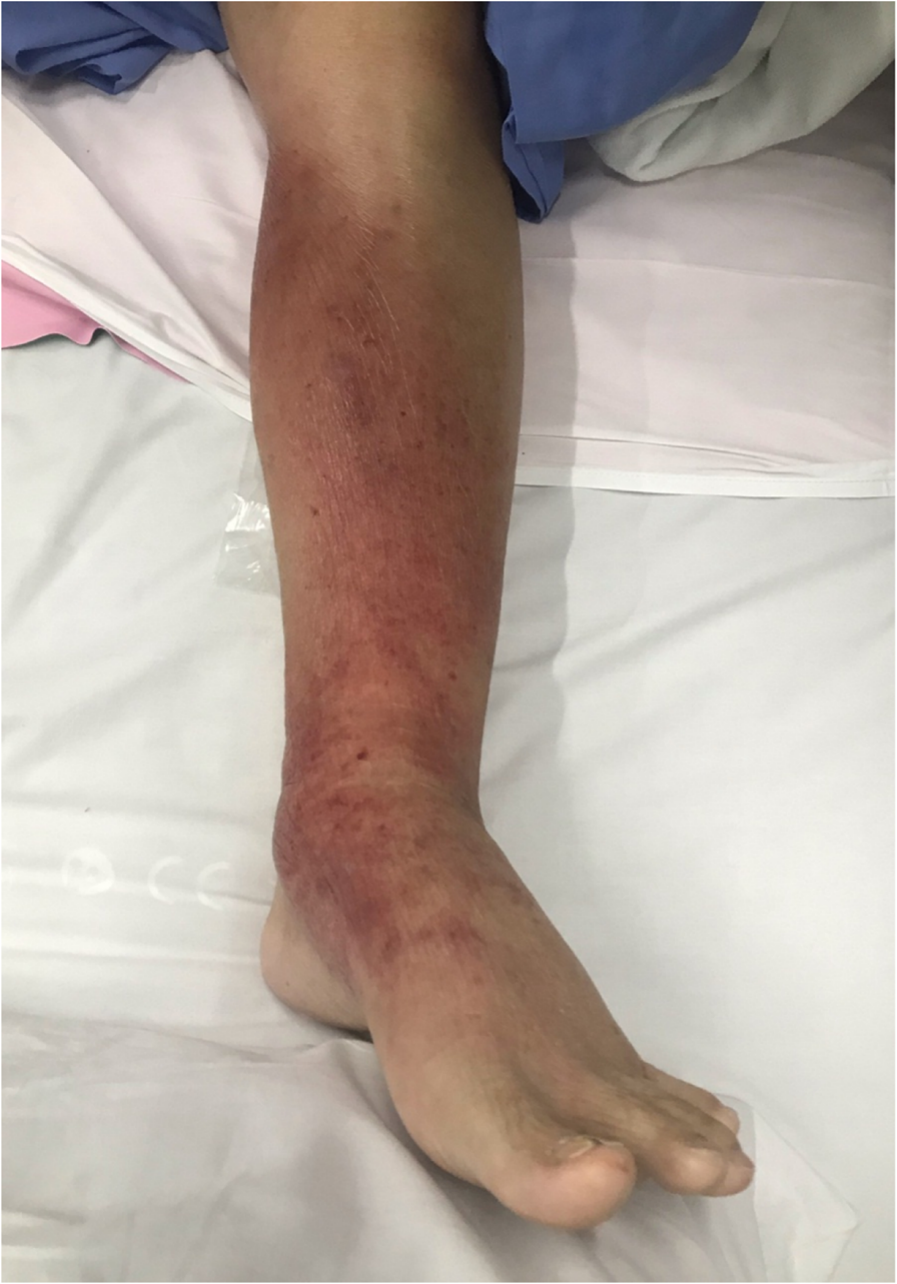
Patient’s left leg showed erythematous lesion with swelling and tenderness

A 70-year-old Thai man with a 5-year history of primary myelofibrosis, hypertension, pulmonary hypertension due to left-sided heart disease, asthma, and osteoporosis presented with an erythematous swollen left leg for 7 days (Fig. 1). He also complained of fever, dyspnea on exertion, and orthopnea for 6 days. He had multiple ulcers on his right foot for 10 days, and was treated with ceftriaxone for 3 days prior to hospitalization. He received ruxolitinib for treatment of primary myelofibrosis for 4 years. His other medications included warfarin, omeprazole, furosemide, diltiazem, spironolactone, bisoprolol, and seretide evohaler for his pulmonary and heart diseases.

Ruxolitinib was initiated in November 2015 after the patient developed pancytopenia following hydroxyurea and allopurinol treatment. Total duration of ruxolitinib therapy was 48 months, and the most recent dose was 20 mg/day. He denied smoking and alcohol drinking. He reported no exposure to herbal drug use or animals, and he had no known tuberculosis contact.

Physical examination revealed a low-grade fever [temperature (T) 38.3 °C], blood pressure (BP) 117/64 mmHg, pulse 72/min, respiratory rate 24/min, oxygen saturation 85% at room air, and 98% with oxygen cannula 3 l per minute (LPM). He was alert, had mild pallor, and was tachypneic. Cardiovascular examination showed jugular vein distension with apical and parasternal heaving, loud P2, and irregular pulse. Fine crepitations were detected at both lower lung fields. Abdominal examination revealed mild splenomegaly. Ill-defined erythematous swelling of the left leg with mild tenderness, and multiple ulcers at his right foot with minimal pus discharge were noted. Neurological examination and other systems were unremarkable.

**Fig. 2:**
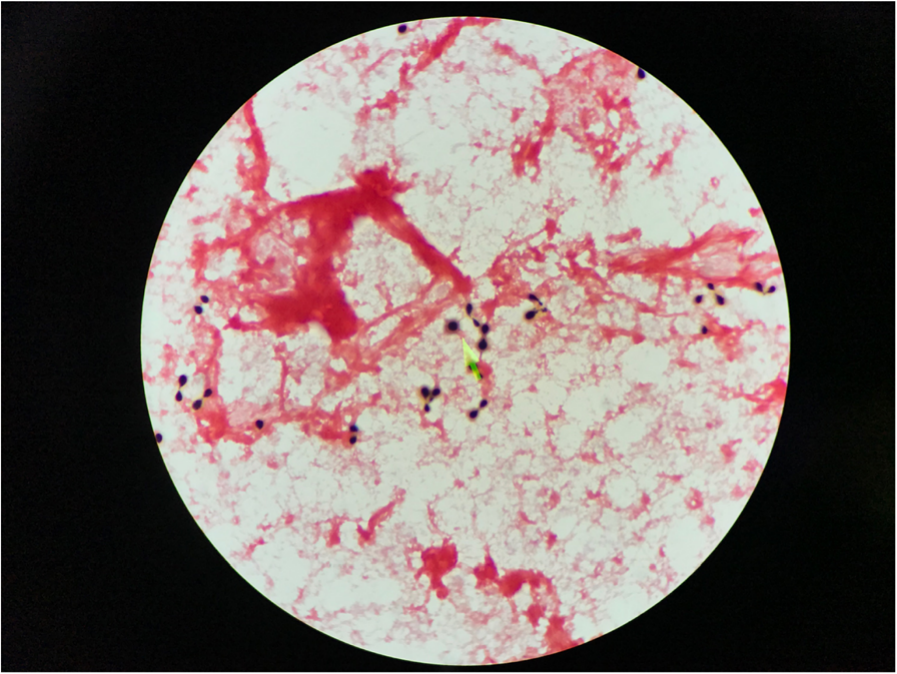
Gram stain from blood culture showed encapsulated budding yeasts

Complete blood count (CBC) revealed a white blood cell count (WBC) of 21,130 cells/μL (81% neutrophil, 9% band form, 5% metamyelocyte, and 2% promyelocyte), hemoglobin (Hb) level of 7.2 g/dL, platelet (PLT) count of 536,000 cells/μL, blood urea nitrogen (BUN) of 23.9 mg/dL, and serum creatinine (SCr) of 1.21 mg/dL. Liver function tests showed direct bilirubin (DBIL) of 0.66 mg/dL, total bilirubin (TBIL) of 0.45 mg/ dL, aspartate transaminase (AST) of 16 U/L, alanine transaminase (ALT) of 10 U/L, alkaline phosphatase (ALP) of 84 U/L, albumin (ALB) of 2.8 g/dL, and total protein of 5.4 g/dL. The urinalysis was unremarkable. Chest radiograph (CXR) showed bilateral interstitial infiltration with blunt costophrenic angles and increased cardiothoracic ratio. One of his 2 blood cultures grew round budding yeasts on the fourth day after blood collection, which were identified as *C. neoformans* (Fig. 2). Serum cryptococcal antigen was positive at a titer of 1:2. Lumbar puncture was attempted; however, no cerebrospinal fluid was collected.

On the admission date, the patient was empirically treated with intravenous ceftazidime and azithromycin. Following the blood culture result, intravenous amphotericin B deoxycholate 50 mg/day (0.87 mg/kg/day) and oral fluconazole 800 mg/day were added. The patient was improved. Subsequently, the patient developed acute kidney injury (AKI) and the antifungal agent was changed to liposomal amphotericin B 180 mg (3 mg/kg/day). The patient received induction therapy with 28 days of intravenous amphotericin B plus fluconazole 800 mg/day, followed by oral fluconazole at the dose of 400 mg/day thereafter. However, ruxolitinib was still continued.

**Fig. 3:**
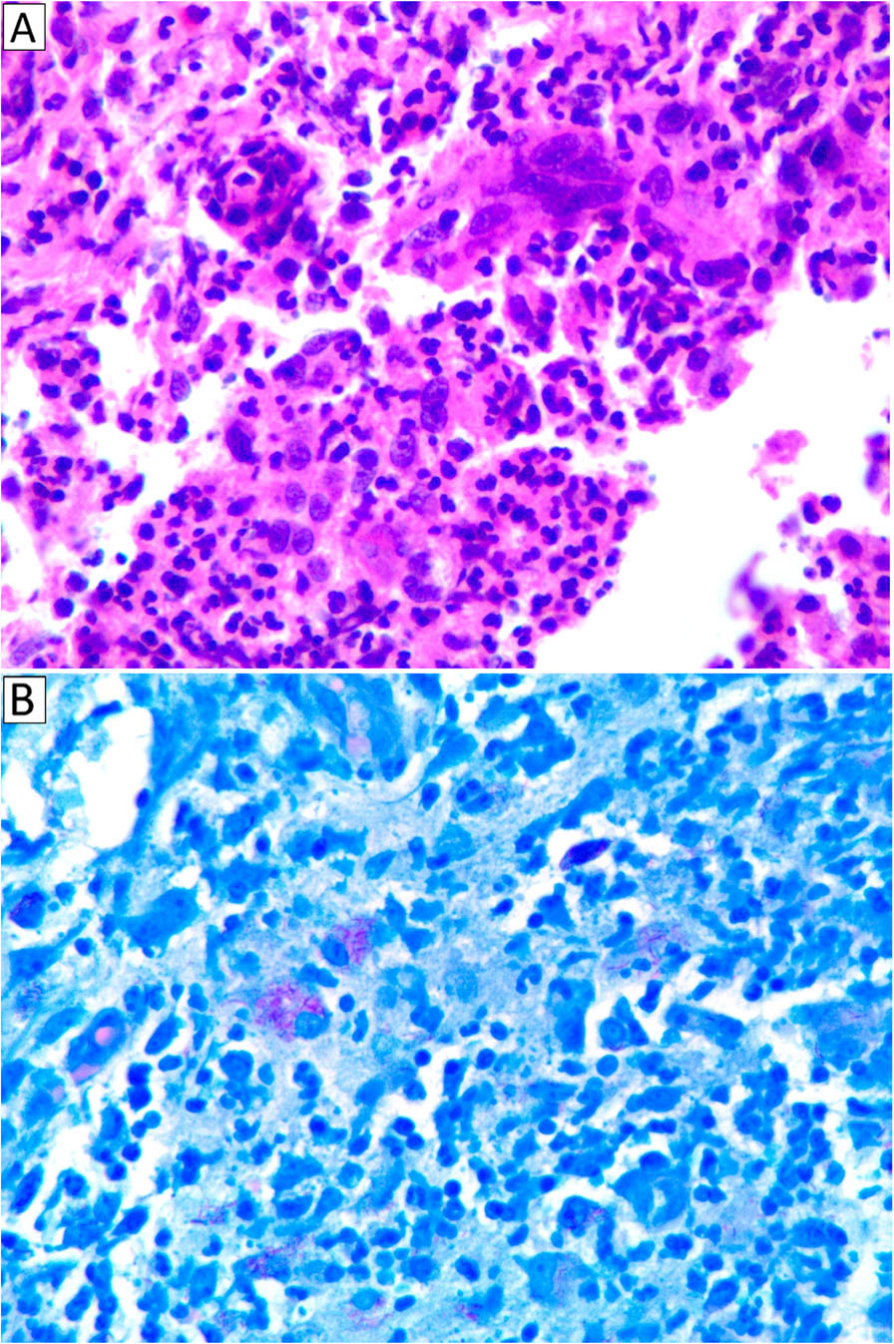
Histopathological findings of skin biopsy from the patient’s left leg revealed suppurative granuloma involving dermis and subcutis (**a)** with numerous acid-fast bacilli visualized by Ziehl-Neelsen staining method (**b**)

After 1 month of hospitalization and antifungal treatment, the patient developed new onset of fever and worsening of the lesion on his left leg. Intravenous vancomycin was initiated for empirical treatment of suspected nosocomial skin and soft tissue infection. Skin biopsy at the left leg lesion was performed. The tissue pathology showed suppurative granuloma involving dermis and subcutis that suggested mycobacterial skin and soft tissue infection, and Ziehl-Neelsen staining demonstrated numerous acid-fast bacilli (Fig. 3). Tissue mycobacterial culture revealed positive organisms from acid-fast stain, and isolate identification by INNO-LiPA assay technique from liquid medium was compatible with *M. haemophilum*. The mycobacterium colony grew on chocolate plate agar after 42 days of incubation, but failed to grow on solid (Lowenstein-Jensen) medium.

The patient was treated with levofloxacin 750 mg/day, rifampicin 300 mg/day, and ethambutol 800 mg/day; however, the patient was not improved after 7 days of antimicrobial treatment. He then developed septic shock and expired on the 48th day of hospitalization.

## Discussion and conclusions

Abnormal regulation of the Janus kinase (JAK) - signal transducer and activator of transcription (STAT) signaling pathway is the major pathophysiology of myelofibrosis. In addition to chemotherapy and hematopoietic stem cell transplantation, many immunomodulatory agents have recently been approved for treatment of this condition [1]. On the other hand, ruxolitinib adversely affects host immune response [2]. The involved immune response components include both innate and adaptive immunity, such as natural killer cells, dendritic cells, T helper cells, regulatory T cells and several cytokines. Therefore, either intracellular or extracellular pathogens can proliferate in the absence of an effective immune response [3].

We searched the term “ruxolitinib AND infection” and “ruxolitinib AND infectious” in the PUBMED database, and there were 59 results of clinical trials, case reports, and literature reviews. From those results, the most common opportunistic pathogens that benefit from these immune defects include bacteria, mycobacteria, viruses, and fungi, similar to those seen in human immunodeficiency virus (HIV)-related infections. The causative pathogens in previously reported cases of ruxolitinib-associated infections are summarized in Table 1 [1–17]. Consistent with the foregoing, it appears that *Cryptococcus* and nontuberculous mycobacteria were able to simultaneously infect our patient due to the downregulation of multiple cytokines.

**Table 1:** Types of organisms and infections in previously reported cases of ruxolitinib-associated infection [1–17]

Organisms	Infections	Number of Patients
Bacteria	Cellulitis/necrotizing fasciitis	1
	*Klebsiella pneumoniae* liver abscess	1
	Bronchitis/pneumonia	NA
	Urinary tract infection	NA
	Osteomyelitis	NA
	Sepsis	NA
Mycobacteria	Tuberculosis	91
	NTM	23
Virus	HSV/VZV infection	4
	HBV reactivation	3
	EBV-related disease (CNS lymphoma, gastric ulcer)	2
	CMV retinitis	1
	Disseminated molluscum contagiosum	1
	JC virus (meningitis, granule cell neuronopathy, PML)	3
Fungus	Coccidioidomycosis	8
	Cryptococcosis	6
	*Pneumocystis jiroveci* pneumonia	3
	Invasive aspergillosis	2
	Disseminated *Talaromyces marneffei* infection	1
	Rhino-orbital mucormycosis	1
Parasite	Toxoplasma retinitis	1

*Abbreviations*: *CMV* Cytomegalovirus, *CNS* Central nervous system, *EBV* Epstein-Barr virus, *HBV* Hepatitis B virus, *HSV* Herpes simplex virus, *NA* Not available, *NTM* Nontuberculous mycobacterium, *PML* Progressive multifocal leukoencephalopathy, *VZV* Varicella zoster virus

From previous case reports of cryptococcal infection, which are summarized in Table 2 [4–6, 9–11], 6 patients with a history of ruxolitinib treatment developed cryptococcal infection. Wysham NG, et al. [4] and Hirano A, et al. [5] reported 2 cases of pulmonary cryptococcosis, but the other four cases presented as fungemia, pericardial involvement, and meningitis or meningoencephalitis. The duration of ruxolitinib treatment was 5–46 months. One patient had a concurrent infection with *Histoplasma capsulatum* [6]. Three of six patients received intravenous amphotericin B deoxycholate for induction therapy, and the other antifungal drugs that were used were fluconazole, voriconazole, flucytosine, and isavuconazole. The outcome was favorable in 5 of 6 patients, and one patient deteriorated during treatment and expired. These observations suggest a benefit of discontinuing ruxolitinib to achieve a good outcome in management of ruxolitinib-associated cryptococcal diseases.

**Table 2:** Literature review for reported cases of cryptococcal infection in patients receiving ruxolitinib

Author [Year]	Patient	Gender/Age	Clinical diagnosis	Underlying disease(s)	Ruxolitinib duration	Symptoms	Laboratory findings	Imaging	Organism/site	Treatment	Outcome
Wysham N, et al. [2013] [4]	1	Male/66	Pneumonia	Polycythemia vera and myelofibrosis	18 months	Dyspnea, cough, and intermittent fever	- Mild leukocytosis - Serum cryptococcal Ag 1:4	Multifocal consolidations with surrounding ground glass opacities	*Cryptococcus neoformans* (BAL)	- Oral fluconazole (5 months) - Discontinue ruxolitinib	Survived
Chen C, et al. [2016] [10]	2	Female/69	Meningoencephalitis	Myelofibrosis	46 months	Fever, alteration of consciousness	- Blood chemistry: NA - CSF cryptococcal Ag 1:512	Normal brain imaging	*- Cryptococcus neoformans* (Fungal CSF culture)	- Amphotericin B and high-dose fluconazole	Survived
Hirano A, et al. [2017] [5]	3	Male/79	Pulmonary nodules	Primary myelofibrosis	6 months	Asymptomatic	- Normal CBC - Serum cryptococcal Ag 1:8	Multiple pulmonary nodules	*Cryptococcus* spp. (TBBx)	- Oral voriconazole (5 months) - Discontinue ruxolitinib	Survived
Liu J, et al. [2018] [9]	4	Male/71	Pericardial effusion	Chronic myelomonocytic leukemia	NA	Cardiac tamponade	- Marked leukocytosis - AKI, metabolic acidosis, liver dysfunction	Bilateral pulmonary infiltrations (CXR)	*Cryptococcus neoformans* (Blood and pericardial culture)	- IV micafungin - IV fluconazole	Died
Prakash K and Richman D [2019] [6]	5	Male/51	Meningitis	Polycythemia vera	18 months	Fever, lethargy, headache, weight loss, oral ulcer	- AKI, hyponatremia, elevated ALP - CSF pleocytosis - Serum cryptococcal Ag 1:128	- Innumerable rim enhancing lesions at the gray-white junction - Retrocardiac mass	*- Cryptococcus neoformans* (Fungal CSF culture) *- Histoplasma capsulatum*	- Amphotericin B and flucytosine - Isavuconazole - Discontinue ruxolitinib	Survived
Tsukui D, et al. [2020] [11]	6	Male/76	Meningitis	Myelofibrosis	5 months	Fever, alteration of consciousness	- Anemia with normal WBC and platelet - CSF pleocytosis - Serum cryptococcal Ag positive (qualitative)	Normal brain imaging	*- Cryptococcus neoformans* (CSF- quantitative assay titers positive 1:16)	- Amphotericin B 37 days, then oral fluconazole - Discontinue ruxolitinib	Survived

*Abbreviations*: *Ag* Antigen, *AKI* Acute kidney injury, *ALP* Alkaline phosphatase, *BAL* Bronchoalveolar lavage, *CBC* Complete blood count, *CSF* Cerebrospinal fluid, *CXR* Chest X-ray, *IV* Intravenous, *NA* Not available, *TBBx* Transbronchial biopsy, *WBC* White blood cell

Ruxolitinib treatment is also associated with mycobacterial reactivation [1, 2, 7]. Case reports of ruxolitinib-associated mycobacterial infections during 2011–2018 are shown in Table 3 [1, 8, 18, 19]. Anand K, et al. reported 91 cases of *M. tuberculosis*, and 23 cases of atypical mycobacterial infections [8]. In previous reports, the site of *M. tuberculosis* infection was identified in 17 cases, with the most common form being disseminated disease, followed by lymphadenitis, pulmonary diseases, and peritonitis [1, 18, 19]. For infections caused by nontuberculous mycobacteria, there were 11 (47.8%) cases of *Mycobacterium avium* complex, 9 (39.1%) unspecified atypical mycobacterium infections, 2 (8.7%) *Mycobacterium kansasii*, and 1 (4.3%) *Mycobacterium abscessus* [8]. Salvator H, et al. reported 1 case of *M. abscessus* lung infection in a 66-year-old female diagnosed with a myelodysplastic syndrome with blastic transformation who underwent allogeneic hematopoietic stem cell transplantation. The patient received ruxolitinib treatment for severe chronic cutaneous graft-versus-host disease, and she developed dyspnea 8 months later. *Mycobacterium abscessus* was identified from lung biopsies. She was treated with imipenem, amikacin, and azithromycin, and ruxolitinib was discontinued. Her symptoms were rapidly improved [20]. To our knowledge, there is no previous case report of *M. haemophilum* infection in a patient treated with ruxolitinib. Here, we report a rare case of primary myelofibrosis treated with ruxolitinib for 4 years who had bloodstream infection with *C. neoformans*, and concurrent cellulitis at his left leg due to *M. haemophilum*.

**Table 3:** Causative organism in reported cases of mycobacterial infection in patients receiving ruxolitinib [1, 8, 18, 19]

Organism	Patients
*Mycobacterium tuberculosis*	91
• Unknown site of infection	74 (81.3%)
• Disseminated tuberculosis	8 (8.8%)
• Tuberculous lymphadenitis	5 (5.5%)
• Pulmonary tuberculosis	3 (3.3%)
• Tuberculous peritonitis	1 (1.1%)
Atypical mycobacterial infections	23
• *Mycobacterium avium complex*	11 (47.8%)
• Unspecified atypical mycobacterium infection	9 (39.1%)
• *Mycobacterium kansasii*	2 (8.7%)
• *Mycobacterium abscessus*	1 (4.3%)

*M. haemophilum* causes skin infections as well as disseminated infection in immunocompromised individuals. It is classified as a nonchromogen by Runyon group classification. Routine microbiological laboratory may report “no growth”, despite the presence of acid fast bacilli in the stain. This is because the bacterium requires heme or iron supplementation in culture media. There is no standard guideline for management of *M. haemophilum* infection, but treatment with a combination of macrolides, fluoroquinolones, and rifampicin has been suggested, with the details of treatment determined on a case-by-case basis [21, 22].

In conclusion, this is the first case of dual infection with *C. neoformans* and *M. haemophilum* in a patient diagnosed with primary myelofibrosis that was being treated with ruxolitinib. This case highlights the possibility of infection caused by multiple atypical pathogens in immunosuppressed patients, especially those receiving ruxolitinib.

## Data Availability

All data generated or analyzed during this study are included in this published article.

## Abbreviations

ALT: Alanine transaminase
ALP: Alkaline phosphatase
AST: Aspartate transaminase
BP: Blood pressure
FDA: Food and Drug Administration
HIV: Human immunodeficiency virus
IL: Interleukin
LPM: Liters per minute
PMF: Primary myelofibrosis
STAT: Signal transducer and activator of transcription
TGF: Transforming growth factor
TNF: Tumor necrosis factor
US: United States

## References

[1] Dioverti MV, Abu Saleh OM, Tande AJ (2018). Infectious complications in patients on treatment with ruxolitinib: case report and review of the literature. Infect Dis (Lond).

[2] Lussana F, Cattaneo M, Rambaldi A, Squizzato A (2018). Ruxolitinib-associated infections: a systematic review and meta-analysis. Am J Hematol.

[3] Elli EM, Baratè C, Mendicino F, Palandri F, Palumbo GA (2019). Mechanisms underlying the anti-inflammatory and immunosuppressive activity of ruxolitinib. Front Oncol.

[4] Wysham NG, Sullivan DR, Allada G (2013). An opportunistic infection associated with ruxolitinib, a novel janus kinase 1,2 inhibitor. Chest..

[5] Hirano A, Yamasaki M, Saito N, Iwato K, Daido W, Funaishi K, Ishiyama S, Deguchi N, Taniwaki M, Ohashi N (2017). Pulmonary cryptococcosis in a ruxolitinib-treated patient with primary myelofibrosis. Respir Med Case Rep.

[6] Prakash K, Richman D (2019). A case report of disseminated histoplasmosis and concurrent cryptococcal meningitis in a patient treated with ruxolitinib. BMC Infect Dis.

[7] Lescuyer S, Ledoux MP, Gravier S, Natarajan-Amé S, Duval C, Maloisel F, Mauvieux L, Toussaint E, Fornecker LM, Herbrecht R (2019). Tuberculosis and atypical mycobacterial infections in ruxolitinib-treated patients with primary or secondary myelofibrosis or polycythemia vera. Int J Infect Dis.

[8] Anand K, Burns EA, Ensor J, Rice L, Pingali SR (2020). Mycobacterial infections with ruxolitinib: a retrospective pharmacovigilance review. Clin Lymphoma Myeloma Leuk.

[9] Liu J, Mouhayar E, Tarrand JJ, Kontoyiannis DP (2018). Fulminant *Cryptococcus neoformans* infection with fatal pericardial tamponade in a patient with chronic myelomonocytic leukaemia who was treated with ruxolitinib: case report and review of fungal pericarditis. Mycoses..

[10] Chen CC, Chen YY, Huang CE (2016). Cryptococcal meningoencephalitis associated with the long-term use of ruxolitinib. Ann Hematol.

[11] Tsukui D, Fujita H, Suzuki K, Hirata K (2020). A case report of cryptococcal meningitis associated with ruxolitinib. Medicine (Baltimore).

[12] Sadjadian P, Wille K, Griesshammer M (2020). Ruxolitinib-associated infections in polycythemia vera: review of literature, clinical significance, and recommendations. Cancers (Basel).

[13] Kusne Y, Kimes KE, Feller FF, Patron R, Banacloche JG, Blair JE, Vikram HR, Ampel NM (2020). Coccidioidomycosis in patients treated with ruxolitinib. Open Forum Infect Dis.

[14] Moruno-Rodríguez A, Sánchez-Vicente JL, Rueda-Rueda T, Lechón-Caballero B, Muñoz-Morales A, López-Herrero F (2019). Invasive aspergillosis manifesting as retinal necrosis in a patient treated with ruxolitinib. Arch Soc Esp Oftalmol.

[15] Sylvine P, Thomas S, Pirayeh E (2018). Infections associated with ruxolitinib: study in French pharmacovigilance database. Ann Hematol.

[16] Ballesta B, González H, Martín V, Ballesta JJ (2017). Fatal ruxolitinib-related JC virus meningitis. J Neuro-Oncol.

[17] Nakayama K, Nakamura M, Konishi A, Kaneko S, Nakamichi K, Saijo M, Yakushiji Y, Kusaka H (2020). JC virus granule cell neuronopathy associated with ruxolitinib: A case report and review of the literature. eNeurologicalSci.

[18] Sakiyama E, Chinen Y, Tsukamoto T, Takimoto-Shimomura T, Kuwahara-Ota S, Matsumura-Kimoto Y, Shimura Y, Kobayashi T, Horiike S, Kuroda J (2020). Tuberculosis peritonitis during treatment of polycythemia vera with ruxolitinib. Infect Drug Resist.

[19] Pepeler MS, Özkurt ZN, Güzel ÖT, Akyürek N (2018). Tuberculosis reactivation related with ruxolitinib in a patient with primary myelofibrosis. J Infect Dev Ctries.

[20] Salvator H, Berti E, Catherinot E, Rivaud E, Chabrol A, Nguyen S, Zemoura L, Cardot E, Tcherakian C, Couderc LJ (2018). Foch Hospital Lung Immune Deficiencies Study Group. Pulmonary alveolar proteinosis and *Mycobacterium abscessus* lung infection related to ruxolitinib after allogeneic stem cell transplantation. Eur Respir J.

[21] Lindeboom JA, Bruijnesteijn van Coppenraet LE, van Soolingen D, Prins JM, Kuijper EJ (2011). Clinical manifestations, diagnosis, and treatment of *Mycobacterium haemophilum* infections. Clin Microbiol Rev.

[22] Nookeu P, Angkasekwinai N, Foongladda S, Phoompoung P (2019). Clinical characteristics and treatment outcomes for patients infected with *Mycobacterium haemophilum*. Emerg Infect Dis.

